# Rare congenital chromosomal aberration dic(X;Y)(p22.33;p11.32) in a patient with primary myelofibrosis

**DOI:** 10.1186/s13039-016-0276-2

**Published:** 2016-08-31

**Authors:** Lenka Pavlistova, Silvia Izakova, Zuzana Zemanova, Lucie Bartuskova, Martina Langova, Pavlina Malikova, Kyra Michalova

**Affiliations:** 1 Center of Oncocytogenetics, Institute of Medical Biochemistry and Laboratory Diagnostics, General University Hospital and First Faculty of Medicine of Charles University, U Nemocnice 499/2, Prague 2, 128 08 Czech Republic; 2 Department of Medical Genetics, Faculty Thomayer’s Hospital, Vídeňská 800, 140 00 Prague 4, Czech Republic; 3 Department of Clinical Hematology IKEM, Vídeňská 1958/9, 140 21 Prague 4, Czech Republic; 4 Department of Cytogenetics, Institute of Hematology and Blood Transfusion, U Nemocnice 1, 128 08 Prague 2, Czech Republic

**Keywords:** dic(X;Y)(p22.33;p11.32), Léri-Weill dyschondrosteosis, Klinefelter syndrome variant

## Abstract

**Background:**

Constitutional translocations between sex chromosomes are rather rare in humans with breakpoints at Xp11 and Yq11 as the most frequent. Breakpoints on the short arm of the Y chromosome form one subgroup of t(X;Y), giving rise to a derived chromosome with the centromeres of both the X and Y chromosomes, dic(X;Y). Here, we report a rare congenital chromosomal aberration, 46,X,dic(X;Y)(p22.33;p11.32)[20]/45,X[10], in an adult male.

**Case presentation:**

Primary myelofibrosis, a malignant haematological disease, was diagnosed in a 63-year-old man following liver transplantation after hepatocellular carcinoma. By the analysis of the bone marrow sample, the karyotype 46,X,dic(X;Y)(p22.33;p11.32) was detected in all the mitoses analysed and verified with multicolour fluorescence in situ hybridization (mFISH). A cytogenetic examination of stimulated peripheral blood cells revealed the constitutional karyotype 46,X,dic(X;Y)(p22.33;p11.32)[20]/45,X[10]. The cell line 45,X was confirmed with FISH in 35 % of interphase nuclei. The *SRY* locus was present on the dicentric chromosome. A CGH/SNP array (Illumina) revealed a gain of 153,7 Mbp of the X chromosome and a 803-kbp microdeletion (including the *SHOX* gene), which were also confirmed with FISH. *SHOX* encodes a transcriptional factor that regulates the growth of the long bones. The deletion of the *SHOX* gene together with the Madelung deformity of the forearm and the short stature of the proband led to a diagnosis of Léri-Weill dyschondrosteosis (LWD). The gain of almost the whole X chromosome (153,7 Mbp) was considered a variant of Klinefelter syndrome (KS). The levels of gonadotropins and testosterone were consistent with gonadal dysfunction. A malformation of the right external ear was detected.

**Conclusions:**

We have reported a structural aberration of the sex chromosomes, dic(X;Y)(p22.33;p11.32). The related genomic imbalance is associated with two known hereditary syndromes, LWD and a KS variant, identified in our proband at an advanced age. Because the breakpoints did not involve cancer genes, we inferred that the two malignancies in the proband were not caused by this abnormality. The possible influence of *SHOX* haploinsufficiency on the growth regulation of auricular chondrocytes is discussed.

## Background

Constitutional translocations between the sex chromosomes are quite rare in humans and are associated with abnormal gonadal development. The ultimate phenotype varies because several other factors are involved: the locations of the breakpoints on the X and Y chromosomes and the corresponding genomic imbalances, the presence/loss of the *SRY* gene locus, tissue mosaicism and an irregular X-inactivation pattern. Breakpoints at Xp11 and of the long arm of the Y chromosome (Yq11), with the loss of the centromere and the *SRY* gene, are the most frequently detected changes. Breakpoints on the short arm of the X and Y chromosomes constitute a rare subgroup of t(X;Y), giving rise to a derived chromosome containing the centromeres of both the X and Y chromosomes, dic(X;Y).

The degree of impairment in the carrier depends on the sex of the individuals and on their sex chromosomes and on the extent of deleted regions on Xp/Yp, which can include genes with variable clinical impacts: ichthyosis (*XLI*), chondrodysplasia punctata (*STS*), ocular albinism (*ARSE*), short stature (*SHOX*), mental retardation (*MRX49*) and Kallman syndrome (*GPR143*). Large deletions of Xp that include many genes and syndromes involving contiguosly deleted genes have been described in males [1, 2]. The *SHOX* gene is localized in pseudoautosomal region 1 (PAR1), which is homologous sequence of nucleotides on both sex chromosomes and comprises 2,6 Mbp at Xp22.33 and Yp11.32. As far as we know, deletions of *SHOX* caused by the formation of dic(X;Y) have been infrequently cited in the literature, with male carriers reported by Wei et al. [3], Mutesa et al. [1], and Mazen et al. [4], and a female carrier of dic(X;Y) with a more proximal breakpoint at Yp11.2 and the loss of the *SRY* gene (determining the male sex) reported by Baralle et al. [5].

Until now, only a few cases of constitutional dic(X;Y), with identical breakpoints at Xp22.33 and Yp11.32, have been described. Familial inheritance is uncommon. McKinley Gardner and Sutherland [6] claimed that this aberration is always sporadic and arises during abnormal X–Y recombination within paternal meiosis. However, the maternal transmission of dic(X;Y)(p22.3;p11.3) was documented in a study by Wei et al. [3]. A woman with the karyotype 45,X/46,X,dic(X;Y)(p22.3;p11.3) gave birth to two children, despite the 80 % 45,X cell line. The contribution of the major 45,X cell line to the female sex is obvious and her fertility was abnormal (she experienced premature ovarian failure at an age of < 30 years). Her son (29 years old at the time of publication) had the karyotype 46,X,dic(X;Y)(p22.3;p11.3)[20]mat, and Léri-Weill dyschondrosteosis (LWD) was diagnosed in both mother and son. The son refused a detailed andrological examination, but his hormonal profile, which included increased levels of luteinizing hormone (LH) and follicle-stimulating hormone (FSH) and reduced testosterone, was consistent with Klinefelter syndrome (KS).

Mazen et al. [4] described *de novo* dic(X;Y)(p22.3;p11.3) in a 14-year old boy with the karyotype 46,X,dic(X;Y)(p22.3;p11.3)[65]/45,X[23]/45,dic(X;Y)(p22.3;p11.3)[12]. The boy had been observed from birth because he was born with ambiguous genitalia, he displayed hypospadias and both testicular and ovarian tissues were detected in his gonads with biopsy. The final diagnosis was ovotesticular disorder of sexual development. DNA sequencing detected a partial deletion of the 5′ region of the homeobox (HMG box) domain of the *SRY* gene. This deletion can reduce the expression of *SRY* and influence its timing and tissue specificity, and therefore testicular development [7]. The consequences of *SHOX* haploinsufficiency and LWD were not discussed in that report. The boy’s growth was not yet complete, but its retardation was already apparent (−2SD), and forearm deformity and wrist pain usually manifest at a later age [8].

*SHOX* is a regulatory gene encoding a transcription factor that plays a key role in bone formation and linear growth, affecting skeletal development and the first and second pharyngeal arches during embryonal development. Its expression pattern parallels the locations of the anatomic structures that are affected when SHOX is deficient: limbs, maxilla, mandible and external ear tract [9]. The heterozygous deletion/mutation of *SHOX* simultaneously with the Madelung deformity of the forearm and a short stature are the main features of LWD. The phenotype is more distinctive in females because oestrogens play a regulatory role in the growth and differentiation of the long bones [8].

## Case presentation

A male patient (born in 1953) suffering hepatocellular carcinoma (HCC), with a significant ethylic aetiology, underwent liver transplantation in October 2014. Six cancer lesions were confirmed in the explant. Because trilinear extra-medullary haematopoiesis was present, he was examined by a haematologist, who diagnosed a myeloproliferative disorder (primary myelofibrosis, PMF). Advanced disease was identified based on the patient’s histology, thrombocytosis of 600–700 × 10^9^/l, and normochronic normocytic anaemia (haemoglobin, 95–110 g/l). Cytoreductive therapy to reduce the number of platelets was indicated and successfully administered up to November 2015. Because the patient’s condition worsened, bone-marrow transplantation was considered, but was postponed because of comorbidity (health status after liver transplantation). A homozygous genomic V617F mutation of JAK2 (a diagnostic molecular marker with a negative prognostic impact that occurs in up to 60 % of PMF) [10] was detected. A cytogenetic analysis of a bone-marrow aspirate was performed.

The bone-marrow cells (collected in November 2014) were cultured for 24 h without mitogen stimulation, and chromosomal preparations were made with standard techniques. At least 20 metaphases were analysed and the karyotype was described according to the International System for Human Cytogenetic Nomenclature (ISCN 2016) [11]. No chromosomal aberrations related to the haematological malignancy were found. (Among the patients with myeloproliferative disorders, the most common chromosomal abnormalities were 20q–, 13q–, +8, +9, 1q+, −7/7q– [12]).

**Fig. 1:**
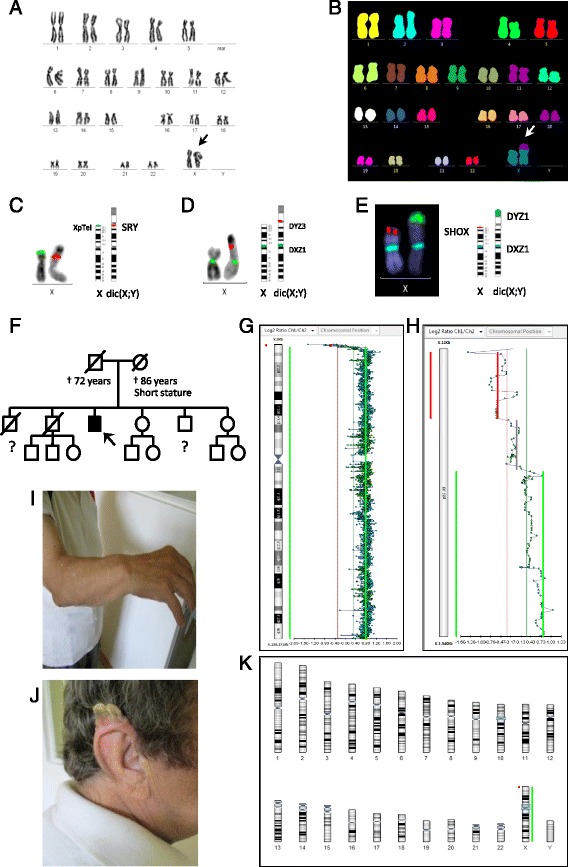
Results of conventional and molecular cytogenetic analyses, clinical genetic examination and documentation of the phenotype. G-banded (**a**) and multicolour FISH karyotypes (**b**) showing dic(X;Y)(p22.33;p13.1). FISH with TelVysion probes Xp/Yp (*green signal*) and LSI SRY (*orange signal*) demonstrating the loss of Xp/Yp subtelomeres on the dicentric chromosome and the present *SRY* gene locus (**c**). FISH with DXZ1 (*green signal*)/DYZ3 (orange signal) (**d**) and deletion of the *SHOX* gene on dic(X;Y) confirmed by FISH with the probe containing the probes for the *SHOX* gene (*orange signal*), DXZ1 (*blue signal*) and DYZ1 (*green signal*) (**e**). Result of CGH/SNP array analysis (**k**). Diagram of the X chromosome (**g**). Chromosomal band Xp22.33 in detail (**h**). Pedigree of the family (**f**). Arrow indicates the affected proband, question mark indicates the unavailable data about the offsprings. Photographs of partial phenotype (**i**, **j**) revealing forearm deformities and auricular hyperplasia

The karyotype 46,X,dic(X;Y)(p22.3;p11.3)[22] was detected in all of the mitoses analysed. This finding was confirmed with multicolour fluorescence in situ hybridization (mFISH) using the 24*X*Cyte mFISH kit (MetaSystems, Altlussheim, Germany) (Fig. 1a and b). FISH performed with commercially available locus-specific probes (Vysis LSI SRY, Vysis TelVysion Xp/Yp) confirmed the deletion of both the subtelomeric regions of Xp/Yp and the present *SRY* gene locus (Fig. 1c).

The dicentric chromosome was identified with FISH and the centromeric probe CEPX (DXZ1)/CEPY (DYZ3) (Fig. 1d). All probes were from Abbott Molecular (Des Plaines, IL, USA).

The constitutional karyotype (from peripheral blood cultured for 72 h with phytohaemagglutinin stimulation) was examined in January 2015. In addition to the 46,X,dic(X;Y)(p22.3;p11.3)[20] translocation, a 45,X[10] cell line was found. Interphase FISH confirmed the 45,X (monosomy X/loss of *SRY* gene) cellular clone in 35 % of nuclei.

Comparative genomic hybridization (CGH) and a single-nucleotide polymorphism (SNP) array (Cytochip Cancer SNP 4 × 180 K; BlueGnome, Illumina, Cambridge, UK) discovered the loss of 803,5 kbp at chromosomal band Xp22.33 (bp 60,726 to 864,243) [the part of pseudoautosomal region 1 (PAR1) including the *SHOX* gene] and a gain of 153,7 Mbp, extending from Xp22.33 to Xq28 (bp 1,518,233 to 155,232,885) (Fig. 1g, h and k).

Result of CGH/SNP array analysis is: 46,X,dic(X;Y)(p22.33;p11.32).arr[GRCh37]Xp22.33(60726_864243)x1,Xp22.33q28(1518233_155232885)x2. PAR1 is graphically displayed only on the X chromosome. Between abnormal regions, still in PAR1, the region of the size 598,8 kbp was evaluated as normal (present in two copies). We assume, that this region is preserved on the Y chromosome and normal X chromosome (deleted on derived X chromosome). The Y chromosome was evaluated as normal. Deletion of the subtelomeric region Yp and the heterozygous deletion of the *SHOX* gene were verified with FISH and Aquarius® Microdeletion Syndrome Probe *SHOX* (Cytocell) (Fig. 1e).

The patient was examined by a clinical geneticist. An examination of his parents is not possible anymore and the available data for other living family members are very limited. The proband’s mother had a short stature and the proband was the smallest of six siblings (Fig. 1f). No intellectual disability was demonstrated. He is stocky (height 152 cm, weight 75 kg) with a massive chest, gynaecomastia, kyphoscoliosis and short limbs (with severely bowed legs). An uncommon auricular malformation was visible on the right ear (Fig. 1j).

The features of the forearms, originating from the bowing radius and the dorsal subluxation of the ulna (Madelung deformity), were obvious (Fig. 1i). LWD was diagnosed based on this feature, the patient’s short stature and the heterozygous deletion of the *SHOX* gene.

The large gain of 153,7 Mbp of DNA, extending from Xp22.33 to Xqter, was shown to involve the gain of almost the whole X chromosome. The presence of an additional derived X chromosome can be considered a Klinefelter syndrome (KS) variant [13]. Despite the microdeletion, the *SRY* gene locus at Yp11.3, which is responsible for male sex determination, was present on dic(X;Y). The tall stature and narrow shoulders that are usually associated with KS are masked by the manifestations of the growth retardation syndrome (LWD). The proband had a short stature and was stubby. Neither syndrome was accompanied by the marked malformations, which typically manifest from birth and/or early childhood. Only his gynaecomastia has been remarkable from puberty. The external genitalia can range from ambiguous to normal male in individuals with KS variant karyotypes. The patient’s external genitalia were not malformed and normal sexual interaction was presumed, as has been reported for other individuals with KS [14].

We assume that the partial deletion of the 5′ region of the HMG box domain of the *SRY* gene was responsible for the severe gonadal malformation in the case described by Mazen et al. [4]. Our patient’s LH, FSH and testosterone levels were determined from a blood sample. Elevated levels of the gonadotropins LH (20.5 U/l; normal range 1.2–8.6 U/l) and FSH (33 U/l; normal range 1.3–19.3 U/l) and reduced levels of testosterone (5.53 nmol/l; normal range 6.10–27.10 nmol/l) are consistent with gonadal dysfunction, and were also detected in the man reported by Wei et al. [3]. Infertility was expected. Currently, cytogenetic analysis is an integral part of the diagnosis in couples with reproductive failure.

Our proband had limited relations with women in the past, and had made no effort to have children. McKinley Gardner and Sutherland [6] stated that male carriers of this translocation are always infertile.

The extent of phenotypic manifestations also depends on cell mosaicism, the proportion of 46,X,dic(X;Y)/45,X cells, and their tissue distributions. The significant clinical impact of the 45,X cell line on the development of different organs has been reported. For example, Kaprova-Pleskacova et al. [15] reported 46,X,psu dic(Y) in a girl with another cell line 45,X and Portnoï et al. [16] described a patient with the mosaic karyotype 45,X/46,X,der(X)t(X;Y)(p11.4;p11.2). In our patient, the *SRY* gene was only present in the dic(X;Y) cells, which constituted 65 % of the cells in the peripheral blood. We infer that *SRY*-positive cells were predominant in the gonadal tissue. The 45,X cell line was not identified by FISH in the bone-marrow cells of our patient. We assume it had a later postzygotic origin in the peripheral blood cells, and did not influence the development of the gonads or other organs.

The malformation of the right external ear after a scissors-inflicted injury during a haircut is very uncommon. However, ear deformities or hearing failure have been reported as the consequences of *SHOX* deletions in women with Turner’s syndrome, although rarely in cohorts of LWD patients [17]. However, to our knowledge, cell dysplasia induced by the healing process of a cut wound has not been reported. The influence of *SHOX* expression on cell proliferation and viability was demonstrated by Marchini et al. [18]. Generally, wild-type SHOX induces cell-cycle arrest and apoptosis by altering the expression of other cell-cycle-regulating proteins in humans (RUNX2, SOX2) [18]. In the growth plate, hypertrophic chondrocytes express SHOX, which exerts a potent antiproliferative effect. A study of in vitro-cultured human auricular chondrocytes confirmed the overexpression of the *RUNX2* gene, which implies hypertrophy [19]. SHOX-mediated cell-cycle arrest is altered when *SHOX* expression is reduced [20], and we hypothesize that the hyperplasia of the auricular cartilage could have been caused in vivo by abnormal levels of cell growth inhibitors, mediated by *SHOX* haploinsufficiency.

The proband suffered two malignant diseases during his life: hepatocellular carcinoma and primary myelofibrosis. The incidence of cancer in carriers of congenital chromosomal aberrations has been extensively studied [21]. *De novo* germ-line genomic changes can target genes that are rearranged in cancer, and the breakpoints of germ-line rearrangements and somatic rearrangements can overlap. The molecular basis and genomic context of sporadic *de novo* rearrangements are not fully understood. The phenotypic outcome may be determined by the timing and context of the rearrangement (involving additional mutations) [22]. The breakpoints in our patient did not affect any cancer genes. The cancer risk of KS cohorts is similar to the general population. Generally, men with KS have an increased risk of specific malignancies: extragonadal germ-cell tumours, non-Hodgkin’s lymphoma and breast cancer [23]. However, the occurrence of multiple cancers has not been equivocally established in individuals with KS. As well as hormonal and genetic factors, factors such as obesity and alcohol abuse also played roles in the development of cancer in our proband.

## Conclusion

The unique case of a very rare congenital translocation between chromosomes X and Y, forming a dicentric chromosome dic(X;Y)(p22.33;p11.32), was detected when the proband was at an advanced age. An analysis of the karyotype in bone-marrow cells, performed to investigate the patient’s haematological malignancy, first detected the two associated syndromes in the proband- LWD and variant KS. Based on data in the literature and the breakpoints involved, the two malignant diseases suffered by the proband are presumed to be unrelated to this inborn chromosomal aberration.

## Abbreviations

CGH/SNP: Comparative Genomic Hybridization/Single-nucleotide Polymorphism
FISH: Fluorescence in situ Hybridization
FSH: Follicle-stimulating Hormone
HCC: Hepatocellular Carcinoma
KS: Klinefelter Syndrome
LH: Luteinizing Hormone
LWD: Léri-Weill Dyschondrosteosis
mFISH: Multicolour Fluorescence in situ Hybridization
PMF: Primary Myelofibrosis
SRY: Sex-determining Region Y (Chromosome)
